# Near-Full-Length Hepatitis B Virus Genomes Isolated from Cerebrospinal Fluid of People Living with HIV and Cryptococcal Meningitis in Botswana

**DOI:** 10.3390/genes17101168

**Published:** 2026-09-23

**Authors:** Basetsana K. S. Phakedi, Bonolo B. Phinius, Kesaobaka Molebatsi, Nametso Kelentse, Lynnette Bhebhe, Linda Mpofu-Dobo, Kabo Baruti, Chanana Tsayang, Graceful Mulenga, Tsholofelo Sethibe, Gorata G. A. Mpebe, Nokuthula S. Ndlovu, Wonderful T. Choga, David S. Lawrence, Sikhulile Moyo, Joseph N. Jarvis, Irene Gobe, Motswedi Anderson, Simani Gaseitsiwe

**Affiliations:** 1 Botswana Harvard Health Partnership, Private Bag BO320, Gaborone, Botswana; bphakedi@bhp.org.bw (B.K.S.P.); bphinius@bhp.org.bw (B.B.P.); kmolebatsi@bhp.org.bw (K.M.); thobanenametso@gmail.com (N.K.); lynnettebhebhe@gmail.com (L.B.); ldobo@bhp.org.bw (L.M.-D.); barutik@ub.ac.bw (K.B.); ctsayang@mail.bhp.org.bw (C.T.); gmulenga@bhp.org.bw (G.M.); tratsoma@bhp.org.bw (T.S.); gmpebe@bhp.org.bw (G.G.A.M.); nsndlovu@mail.bhp.org.bw (N.S.N.); wchoga@bhp.org.bw (W.T.C.); david.s.lawrence@lshtm.ac.uk (D.S.L.); smoyo@bhp.org.bw (S.M.); joseph.jarvis@lshtm.ac.uk (J.N.J.); manderson@bhp.org.bw (M.A.); 2 School of Allied Health Professions, Faculty of Health Sciences, University of Botswana, Private Bag UB 0022, Gaborone, Botswana; gobei@ub.ac.bw; 3 Department of Biological Sciences and Biotechnology, School of Life Sciences, Botswana International University of Science and Technology, Private Bag 16, Palapye, Botswana; 4 Department of Mathematics and Statistical Sciences, Faculty of Science, Botswana International University of Science and Technology, Private Bag 16, Palapye, Botswana; 5 Department of Biological Sciences, Faculty of Sciences, University of Botswana, Private Bag UB 0022, Gaborone, Botswana; 6 Department of Clinical Research, Faculty of Infectious and Tropical Diseases, London School of Hygiene and Tropical Medicine, London WC1E 7HT, UK; 7 School of Pathology, Faculty of Health Sciences, University of the Witwatersrand, Johannesburg 2193, South Africa; 8 Department of Immunology and Infectious Diseases, Harvard T.H. Chan School of Public Health, Boston, MA 02115, USA; 9 Division of Virology, Department of Pathology, Stellenbosch University, Cape Town 7505, South Africa; 10 School of Health Systems and Public Health, University of Pretoria, Pretoria 0002, South Africa; 11 Africa Health Research Institute (AHRI), Durban 4043, South Africa; 12 The Francis Crick Institute, London NW1 1AT, UK

**Keywords:** hepatitis B virus, cerebrospinal fluid, HIV, cryptococcal meningitis, central nervous system, viral compartmentalization, Botswana

## Abstract

**Background/Objectives:** Increasing evidence suggests that the hepatitis B virus (HBV) can be detected in extrahepatic compartments. Data on the molecular characteristics of HBV in cerebrospinal fluid (CSF) remain limited, particularly among people living with HIV (PLWH) and cryptococcal meningitis (CM). This study aimed to characterize HBV genomes from plasma and CSF and compare paired sequences for genetic similarity and potential compartment-specific differences. **Methods:** Archived plasma and CSF samples from PLWH with CM from Botswana enrolled in AMBITION-CM trial (2018–2021) were sequenced using Oxford Nanopore Technology to generate near-full-length HBV genomes. Genotyping and mutational analysis were performed and paired plasma–CSF sequences were compared. **Results:** Four sequences were generated, including one plasma–CSF pair and two CSF-only sequences. The sequences clustered with genotypes A1 and D3. The single paired plasma–CSF sequences shared 98.8% nucleotide similarity, and clustered together with strong phylogenetic support (SH-aLRT/ultrafast bootstrap = 99.6/100). Variant analysis identified 492 and 545 high-quality variants (Q ≥ 30) in plasma and CSF, respectively. In the single paired sequences, hepatocellular carcinoma (HCC)-associated mutation T1858C was detected in plasma but not in the CSF. Mutations were also identified in CSF-derived sequences, including A1762T/G1764A, G1896A, G1899A, associated with increased HCC risk, rtS184L, rtF194L, rtP236T, linked to antiviral resistance and sP127L, sQ129L, sM133L, sS136P, associated with immune escape. **Conclusions:** Near-full-length HBV genomes were successfully recovered and genetically characterized from the CSF of PLWH with CM. The single plasma–CSF paired sample, showed high genetic similarity and had a compartment-specific mutation. Clinically relevant mutations were observed in the CSF warranting further investigation of HBV in the CSF to confirm these observations in PLWH with CM. These preliminary findings from a limited number of participants provide a basis for larger-scale studies of near-full-length HBV genome characterization in CSF.

## 1. Introduction

Hepatitis B virus (HBV) is classically regarded as a hepatotropic virus, but increasing evidence shows that HBV can also be detected in extrahepatic compartments [1,2,3,4]. Reports of hepatitis B surface antigen (HBsAg) and HBV DNA in cerebrospinal fluid (CSF), together with detection in brain tissue, have raised the possibility that the central nervous system (CNS) may be involved in HBV dissemination in selected clinical settings [3,4,5]. HBV primarily infects hepatic cells through the sodium taurocholate co-transporting polypeptide (NTCP) receptor, which is highly expressed in liver cells; however, in the central nervous system, it remains unclear whether HBV replication occurs, as there are no well-established cell types known to express the NTCP receptor [6].

During replication, viral particles and antigens are released into the bloodstream, making blood a medium for diagnosing and monitoring HBV infection. Accordingly, blood or plasma remains the routine specimen used for HBV diagnosis, virological monitoring, and treatment decision-making in clinical practice and guideline-based care [7]. Detecting HBV in the CNS may suggest hematogenous transport of viral particles to the brain; however, the Blood–Brain Barrier (BBB) is believed to be able to limit the entry of viral particles into the CNS [8]. Neuropathies may be a leading cause of viral entry as they disrupt the BBB, allowing for viral particles to pass through. People living with HIV (PLWH), particularly those with advanced HIV disease (AHD), are highly susceptible to CNS opportunistic infections or neuropathies such as cryptococcal meningitis (CM) [9]. CM, caused by the Cryptococcus species, induces inflammation and increased intracranial pressure, both of which contribute to structural and functional disruption of the BBB [10]. Such inflammation-mediated BBB dysfunction may increase permeability, potentially allowing circulating viruses, including HBV, to access the CSF. Once HBV is within the CNS, HBV may persist and potentially undergo localized evolution distinct from viral populations replicating in hepatocytes.

The presence of HBV in the CSF raises the possibility that the virus may establish a distinct niche within the CNS. A previous study has reported substantial genetic diversity between paired plasma and CSF HBV sequences, suggesting possible viral compartmentalization [4]. In the CNS, the BBB limits drug penetration and the CNS is characterized by low immune pressure, which may allow HBV viral persistence and emergence of adaptive mutations associated with drug resistance or vaccine escape [8]. Such evolution could potentially contribute to viral persistence, reinfection following liver transplant, and long-term complications such as cirrhosis and hepatocellular carcinoma (HCC). This potential evolution is relevant in PLWH with advanced disease, where CNS opportunistic infections are common. CM, caused by Cryptococcus neoformans, is characterized by CNS invasion and inflammatory injury, and the pathogen is known to breach the BBB through multiple mechanisms [11,12]. This disruption of BBB integrity may facilitate viral entry into the CNS or influence viral dynamics within this compartment. Despite reports of HBV detection in CSF, limited information on HBV compartmentalization exists. Strong evidence of HBV replication within the CNS is lacking, and it remains unclear whether HBV detected in the CSF represents true compartmentalized replication, passive spillover from high plasma viral load, transient neuroinvasion, or the presence of a distinct compartment-associated viral population that is better suited for CNS invasion. Understanding HBV presence and compartmentalization in the CNS is important for HBV control and elimination efforts. If the CNS serves as a potential viral reservoir, it may represent a site of ongoing replication and evolution that is not fully captured by plasma screening and monitoring. Therefore, this study aimed to characterize near-full-length HBV genomes recovered from CSF and compare paired plasma–CSF sequences to explore any genetic differences detected between the two compartments in people living with HIV-associated cryptococcal meningitis in Botswana.

## 2. Materials and Methods

### 2.1. Study Population

This was a retrospective cross-sectional study utilizing archived baseline plasma and CSF samples collected from participants enrolled at the Botswana site for the AMBITION-CM (AmBisome Therapy Induction Optimization) Phase 3 randomized controlled trial, conducted between 2018 and 2021 (ISRCTN: 72509687) [13]. Briefly, the trial compared a single high dose of liposomal amphotericin B combined with 14 days of flucytosine and fluconazole to the WHO-recommended first-line regimen of one week of amphotericin B deoxycholate plus flucytosine, followed by one week of fluconazole. This cohort represented the only available plasma–CSF specimens suitable for this retrospective analysis. Ten adults living with HIV and diagnosed with CM in the AMBITION-CM study who had available archived residual paired plasma and CSF samples at baseline were included in this study. Ethical approval was obtained from the University of Botswana Institutional Review Board and the Health Research and Development Council at the Botswana Ministry of Health (HRDC#01057). All participants consented to the future use of their biological samples.

### 2.2. Extraction of Plasma or CSF Samples

HBV DNA was extracted from 200 µL of plasma and CSF samples that were positive for HBsAg or with detectable HBV DNA from an earlier study using the QIAamp DNA Blood Mini Kit (Qiagen, Hilden, Germany) [14]. The extraction process followed the manufacturer’s protocol, with a final elution volume of 30 µL to concentrate the extracted DNA.

### 2.3. Amplification and Sequencing

A near-full-length 3 kb HBV fragment was amplified by nested PCR using PrimeSTAR^®^ GXL DNA Polymerase (Takara Bio Inc., Kusatsu, Japan) with 5 µM of P1 and P2 primers for the first round and 5 µM P3WRS and P4WRS primers for the second round, in a 25 µL total reaction as previously described by Anderson et al., 2018 [15]. The amplicons were then visualized on a 1% agarose gel stained with ethidium bromide, and successfully amplified 3 kb fragments were carried forward for sequencing. HBV amplicons were prepared for sequencing using the Rapid Barcoding Sequencing Kit 96 (SQK-RBK114.96; Oxford Nanopore Technologies, Oxford, UK), following the manufacturer’s protocol as previously described [16]. Prepared libraries were loaded onto R10.4.1 flow cells (FLO-MIN114) and sequenced on the GridION platform (Oxford Nanopore Technologies, Oxford, UK). Base calling and demultiplexing were performed in high-accuracy mode using MinKNOW v26.01.21. The resulting FASTQ files were exported and further processed with Guppy v6.5.7 using dual-indexed read demultiplexing. Reference-based assembly of HBV genomes was performed using HBVgenomer v2.0, an in-house Python-based analysis tool. Assembled consensus sequences were downloaded in FASTA format for downstream phylogenetic and mutational analyses.

### 2.4. Phylogenetic Analysis and Genotyping

Approximately 3 kb of HBV sequences generated in this study were aligned using AliView v1.28 (https://doi.org/10.1093/bioinformatics/btu531) with reference sequences from Botswana retrieved from the National Center for Biotechnology Information (NCBI) GenBank database (https://www.ncbi.nlm.nih.gov/labs/virus/) (accessed on 2 May 2026). The sequences were subsequently trimmed to the same length. Phylogenetic analysis was then performed using a maximum-likelihood approach. The resulting HBV nucleotide sequence alignments were used to infer phylogenetic trees with IQ-TREE v3. Branch support was assessed using the approximate likelihood-ratio test (aLRT) with 1000 replicates and ultrafast bootstrap analysis with 1000 replicates. HBV genotypes were initially assigned based on clustering reference sequences of known genotypes in the phylogenetic tree and subsequently confirmed using the geno2pheno (HBV) online database (https://hbv.geno2pheno.org) (accessed on 2 May 2026).

### 2.5. Pairwise Sequence and Ambiguous Nucleotide Comparison

Matched plasma–CSF HBV consensus sequences were analyzed in R (version 2025.05.0+496). Pairwise sequence comparisons were performed after alignment in AliView with pairwise deletion of gaps and missing data. Nucleotide differences between paired sequences were identified by comparing aligned nucleotide positions and noting sites that differed between compartments at the same nucleotide position. The total number of differing positions was calculated and used to determine sequence similarity between plasma and CSF sequences.

Ambiguous nucleotide positions were identified based on IUPAC ambiguity codes present in consensus sequences generated using a 5% variant-frequency threshold. Positions containing ambiguous nucleotides in aligned sequence were noted and compared between plasma and CSF compartments.

### 2.6. Mutational Analysis

Mutational analysis was performed using HBVgenomeR (v5.9.7) [17]. Consensus HBV genome sequences were screened for clinically relevant mutations associated with antiviral resistance, immune escape, vaccine escape, diagnostic escape, and HCC risk. Drug-resistance analysis was conducted using the HBVgenomeR resistance interpretation module, which performs reverse-lookup of documented HBV resistance mutations across five antiviral agents: lamivudine (LAM), entecavir (ETV), adefovir (ADV), tenofovir (TDF), and telbivudine (LdT). Detected mutations were annotated according to codon position, amino acid substitution, sequencing depth, variant frequency, and predicted resistance level. Immune and vaccine-escape mutations were assessed through analysis of the HBsAg major hydrophilic region (MHR), including mutations associated with vaccine escape, diagnostic escape, and immune selection pressure. Additional screening was performed for mutations associated with HCC, including basal core promoter (BCP) and precore mutations.

## 3. Results

### 3.1. Participant Characteristics and PCR Amplification Success

Amplification was attempted on 17 samples from 10 participants, comprising seven plasma samples and 10 CSF samples. Of the 17 samples, 14 comprised seven plasma–CSF pairs and three were CSF-only samples (Table A1). Successful amplification of the 3 kb HBV fragment was achieved in four of the 17 samples (23.5%), which included one plasma–CSF pair which was assigned pair 1 from participant 1 and denoted P1, and two single CSF-only samples from participant 2 and 3 denoted as P2 and P3 respectively. All participants were male with ages ranging from 29 to 45 years and were all living with HIV-associated CM. The three participants demographics are listed in Table 1 and Table A1.

### 3.2. Sequence Comparison Between CSF and Plasma Compartments

#### 3.2.1. Plasma–CSF Pair Sequence Comparison

All four samples were successfully sequenced. Variants were identified from sequenced reads and variants with quality scores ≥ 30 were used for analysis. For the P1 plasma–CSF pair, sequencing generated mean depths of 7960× and 1945× in plasma and CSF, respectively, with genome coverage exceeding 97% in both samples. Pairwise comparison of the P1 plasma and CSF HBV sequences showed 98.8% similarity at 5% consensus level, with only 37 differences out of 3197 comparable positions.

#### 3.2.2. HBV Variants in P1

Variant analysis using HBVgenomeR identified 492 and 545 high-confidence nucleotide variants relative to the reference HBV genome used (FJ692557) in the plasma and CSF sequences, respectively. All variants included in the analysis had sequencing quality scores Q ≥ 30.

### 3.3. Phylogenetic Analysis

A maximum-likelihood phylogenetic tree was constructed from a 2797 bp alignment of the four study sequences together with reference sequences of known genotypes. The pair P1’s plasma and CSF sequences clustered together on the same branch with high statistical support (SH-aLRT/ultrafast bootstrap = 99.6/100), as shown in Figure 1. The P1 sequences clustered closely with genotype A1 reference sequences. The genotypes were confirmed in geno2pheno and HBVgenomeR outputs and were consistent with genotype A1. Figure 1 also shows that the two CSF-only sequences, P2 and P3, each formed a separate cluster with genotype D3 references, suggesting they both belong to genotype D3. The genotypes were also confirmed in geno2pheno and HBVgenomeR.

### 3.4. Mutation Profile

Clinically relevant HBV mutations were identified in the sequences generated in this study. HBV drug-resistance, vaccine-escape, and HCC-associated mutations were assessed in all samples. In participant P1, no drug-resistance or vaccine-escape mutations were detected in either compartment. However, the HCC-associated mutation T1858C was identified in the plasma sequence but was absent in the matched CSF sequence, indicating mutational discordance between compartments. The P2 CSF sequence contained the basal core promoter double-mutation A1762T/G1764A together with the precore mutations G1896A and G1899A, mutations that have been associated with the risk of HCC. The P3 CSF sequence contained polymerase substitutions rtS184L, rtF194L, and rtP236T, associated with drug resistance, as well as surface antigen substitutions sP127L, sQ129L, sM133L, and sS136P, associated with immune escape (Table 2).

## 4. Discussion

In this study, HBV was successfully isolated, amplified and sequenced from CSF samples. HBV full genomes have been isolated from the CSF compartment and have been reported in previous studies [4,18]. A few studies have also characterized HBV genomes isolated from CSF, and even fewer have compared paired plasma and CSF sequences. Although previous studies have demonstrated recovery of HBV genomes from CSF, data from the African region remain extremely limited. To our knowledge, this study provides the first molecular characterization of near-full-length HBV genomes recovered from CSF in PLWH with CM in the WHO African Region, particularly in Botswana. Studies in Botswana have comprehensively isolated and characterized HBV from plasma with predominantly circulating strains being genotype A1 and D3 [19,20,21]. The detection and isolation of HBV within the CSF supports previous observations that HBV may be present in extrahepatic compartments and raises questions regarding the potential role of the CNS as a viral reservoir especially in high immunocompromised populations with CNS disorders.

Participant 1 (P1) provided a single plasma–CSF paired comparison from near-full-length plasma and CSF sequences which were available across a large overlapping region. In this participant, the paired plasma and CSF sequences clustered closely together with strong statistical support (SH-aLRT/ultrafast bootstrap = 99.6/100). Similarly, pairwise nucleotide analysis showed high similarity of 98.8% between plasma and CSF-derived HBV sequences, indicating these paired sequences are very closely related. The two sequences clustered closely together and formed a distinct branch within the genotype A1 clade, clustering with A1 reference sequences. Consistent with phylogenetic analysis, both sequences were classified as genotype A1 by geno2pheno and HBVgenomeR. Genetic concordance between pairs has been observed in previous studies where sequences from different compartments from the same participant showed concordant genotypes and close clustering of paired plasma and CSF HBV sequences [4]. However, Xu et al., 2023, also reported two out of 10 paired CSF–serum samples having discordant genotypes which differs from our findings [4]. Our findings suggest that in this participant (P1), the viral populations in CSF appear to be closely related to those circulating in the blood based on phylogenetic clustering. These finding should be considered an observation from a single case rather than evidence of a general pattern of HBV population structure between plasma and CSF. A larger cohort is needed to investigate if this observation and pattern is consistently observed with a larger sample size and multiple paired samples with a longitudinal nature.

Ambiguous nucleotide positions were observed in the paired plasma–CSF sequences at the 5% variant-frequency threshold. Of the 130 ambiguous positions identified, 75.0% occurred at similar positions in both plasma and CSF sequences. However, because only one matched pair was available and haplotype reconstruction or longitudinal sequencing was not performed, these observations were not used to infer intra-host viral diversity or HBV population structure between the compartments. Sequencing-related uncertainty may also have contributed to some ambiguous nucleotide calls. Therefore, these findings are reported descriptively and should be interpreted with caution [22].

In the paired P1 sample, the HCC-associated mutation T1858C was detected in plasma but not in the CSF sequence, representing a compartment-specific observation in this particular individual. However, a single paired comparison cannot establish differential mutational distribution between compartments, highlighting the need for larger studies to confirm this observation and determine its clinical significance. T1858C has been associated with more advanced liver disease and an increased risk of HCC [23]. The absence of this mutation in the paired CSF sequence, despite the overall high nucleotide similarity between compartments, raises questions around the possibility of clinically relevant variants being differentially represented in plasma and CSF. Although only one paired plasma-CSF comparison was done, the biological significance of this finding warrants further investigation in larger cohorts. Similar compartment-specific HBV mutations have previously been reported in paired plasma–CSF studies [5]. Although the mechanisms underlying these observations remain unclear, these consistent reports of mutational discordance between plasma and CSF variants warrant further investigation to determine whether it reflects intra-host viral diversity, differences in the relative abundance of viral variants, or compartment-specific evolutionary processes. The P1 plasma sequence generated in the present study was also compared with a previously generated sequence (BKSP010) from the same plasma specimen in an earlier study [14]. In the previous study, amplification and sequence targeting of a 415 bp fragment covering the surface/polymerase region was reported. The corresponding region of the larger sequence generated in the present study was subsequently trimmed to the same region, producing a 350 bp overlap for direct comparison. The two sequences showed complete concordance in genotype and mutational profile across the overlapping region, with no differences identified. This concordance provides additional confidence in the reproducibility and accuracy of the P1 plasma sequence generated in the present study. However, this was the only specimen shared between the two studies.

While the paired P1 plasma–CSF comparison provided an opportunity to examine the relationship between HBV sequences across compartments, the two CSF-only sequences (P2 and P3) provided additional descriptive information on the HBV genomes recovered from CSF. Both sequences clustered with genotype D3 reference sequences, with genotype confirmation from geno2pheno and HBVgenomeR. This genotype is commonly reported in Botswana [19,20,21]. The two genotype D3 CSF sequences each demonstrated distinct mutational profiles.

The P2 CSF-derived sequence contained the basal core promoter double-mutation profile A1762T/G1764A together with the precore mutations G1896A and G1899A. These mutations have previously been associated with altered viral replication, reduced HBeAg expression, progressive liver disease, and an increased risk of HCC [24,25]. In P2, the plasma sample was HBsAg-negative with very low detectable HBV DNA, raising the possibility of an occult HBV infection (OBI) profile [26]. Absence of detectable HBsAg in plasma may be caused by altered antigenicity of the HBV surface protein. Mutations within the major hydrophilic region (MHR), particularly the immunodominant “a” determinant, have been described in OBI and can reduce recognition of HBsAg by immunoassays, potentially contributing to HBsAg detection failure despite the presence of HBV DNA [27,28]. However, in the absence of paired plasma samples and with the lack of comparative sequences of the surface gene, it cannot be confirmed if the plasma sample was a true OBI and if the discordance in HBsAg is a reflection of different antigenicity between the compartments.

In the previous study, plasma and CSF samples were diluted prior to HBV DNA testing because of limited sample volume, which may have reduced the effective concentration of viral DNA that may have affected viral-load quantification or detection. Requantification of diluted samples was not possible because of insufficient remaining sample volume. This is particularly relevant in OBI, where HBV DNA levels are characterized by being low and may approach the analytical detection limit of molecular assays [14,29]. Therefore, the apparent difference in HBV DNA detection between plasma and CSF should be interpreted cautiously, as the prior dilution of the CSF sample may have contributed to the reduced detectability of HBV DNA. The HBsAg assay was not subject to the same dilution procedure but warrants further investigation to observe antigenicity patterns between compartments. Successful HBV sequencing has previously been reported from samples with HBV DNA below the limit of detection of quantitative assays, demonstrating that failure to detect HBV DNA by routine viral-load testing does not necessarily preclude successful amplification and sequencing [20]. Despite these discrepancies, HBV was successfully amplified from CSF with undetectable HBV DNA, highlighting the need for further investigation of clinically relevant HBV variants that may be detectable in CSF even when circulating HBV DNA levels are low or undetectable. Similar observations were reported by Xu et al. (2023), who recovered full-length HBV genomes from CSF despite serum HBV DNA levels being very low, at <20 IU/mL, supporting the possibility that HBV may persist within extrahepatic compartments even when circulating viral markers are absent or present at very low levels [4].

Similarly, the P3 CSF-derived sequence contained a distinct set of polymerase and surface antigen mutations. Polymerase substitutions rtS184L, rtF194L and rtP236T have been associated with reduced susceptibility to antiviral therapy, while the surface antigen substitutions sP127L, sQ129L, sM133L and sS136P occur within or near the major hydrophilic region of HbsAg, which is known to contain vaccine-escape and immune-escape mutations [30]. However, because only qualitative HBsAg results were available and anti-HBs measurements were unavailable, the clinical significance of these mutations could not be extensively evaluated in this cohort. The identification of these mutations in a CSF-derived HBV genome, however, further highlights the need to further investigate genetic diversity of viral populations that may be present within the CNS compartment.

## 5. Limitations

This study has several limitations. First, the small sample size and the low number of successfully amplified paired plasma–CSF samples limited the statistical power of the study. Near-full-length HBV genomes were recovered from only three participants, with only one paired plasma–CSF genome available for comparison. This made it difficult to assess HBV compartmentalization or draw conclusions about independent viral evolution or persistence in the CNS. Therefore, these findings should be considered preliminary and need to be confirmed in larger studies.

Another limitation of the study was the limited sample volume available for some plasma and CSF samples, which required dilution before HBV DNA testing. Although dilution-adjusted HBV DNA concentrations were calculated, repeat testing using undiluted samples was not possible because of insufficient residual sample volume. Therefore, the potential effect of dilution on HBV DNA detection and quantification could not be independently assessed. This may be particularly important for samples with low-level viraemia or low HBV DNA, such as those with OBI, where HBV DNA levels may be close to the assay’s limit of detection. To assess the possibility of contamination, negative controls were included during DNA quantification. Negative controls were also included during DNA extraction, PCR and sequencing.

Successful amplification of the approximately 3 kb HBV genome depended not only on viral load but also on DNA quality. The use of archived residual samples, which may have undergone multiple freeze–thaw cycles, could have reduced DNA quality and contributed to the low amplification success rate. This may explain why some samples with high HBV DNA levels did not amplify successfully. Future studies should use larger sample sizes and freshly collected samples to improve amplification and sequencing success rates.

Oxford Nanopore sequencing has a higher error rate than short-read sequencing technologies. Although high-accuracy base calling, quality filtering (Q ≥ 30), and high sequencing depth were used to improve sequence accuracy, some ambiguous nucleotide calls, low-frequency variants, and small insertions or deletions may have resulted from sequencing errors. Therefore, these findings should be interpreted with caution.

Although long-read sequencing allows haplotype reconstruction and quasispecies analysis, the small number of successfully amplified samples meant that only consensus genome analysis was performed. Future studies with larger sample sizes and deeper sequencing should include haplotype reconstruction and quasispecies analyses to better understand HBV diversity within and between compartments.

Finally, this study did not determine whether HBV detected in the CSF represented active viral replication in the CNS or passive entry from plasma. Markers of active replication, such as HBV pregenomic RNA (pgRNA) and covalently closed circular DNA (cccDNA), were not measured. In addition, HBeAg, anti-HBs, and quantitative HBsAg data were unavailable, making it difficult to assess the clinical significance of some mutations. Future studies should include these analyses to better understand the role of the CNS as a potential HBV reservoir.

## 6. Conclusions

This pilot study demonstrates the feasibility of detecting and molecularly characterizing near-full-length HBV genomes from the CSF of people living with HIV-associated CM. HBV genomes recovered from the three participants belonged to genotypes A1 and D3, consistent with the genotypes circulating in Botswana. In the single participant with paired plasma–CSF genomes, the sequences showed a high genetic similarity, clustering together with strong statistical support. Clinically relevant mutations associated with HCC risk, antiviral resistance, and immune escape in CSF-derived HBV genomes and a mutational discordance between plasma and CSF call for further investigations to confirm these observations. These findings describe the HBV genomes recovered in this small cohort and call for larger studies incorporating multiple pairs, longitudinal plasma–CSF samples and complementary markers of viral replication to determine the biological significance of HBV detected in CSF and potentially establish HBV compartmentalization, independent CNS evolution, or persistence within the CNS.

## Figures and Tables

**Figure 1:**
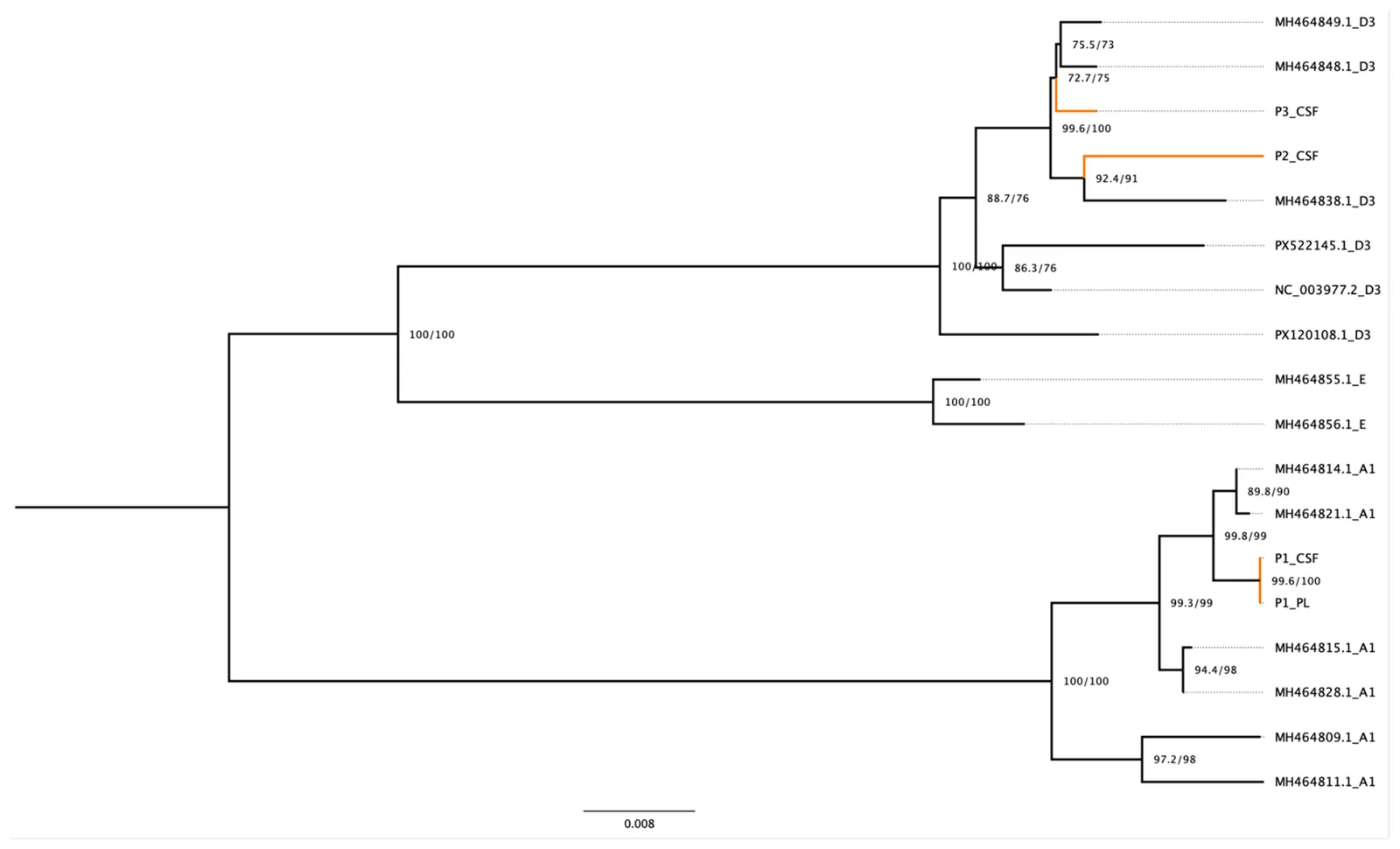
Maximum-likelihood phylogenetic tree of HBV sequences from plasma and CSF, based on a 2797 bp overlap. Sequences generated in this study are indicated in red, while reference sequences representing genotypes A1, D3, and E are included for comparison and indicated in black. Node support values are shown as SH-aLRT/ultrafast bootstrap percentages.

**Table 1:** Individual baseline demographics, clinical and HBV virological characteristics of participants (*n* = 3) with successful amplicons.

PID	Age (Years)	Sex	Mental Status	CSF WBC (Cells/µL)	Plasma HBsAg	Plasma HBV VL (IU/mL)	CSF HBsAg	CSF HBV VL (IU/mL)	Genotype
P1	37	M	Normal	2	+	1.7 × 10^8^	+	142,192	A1
P2	29	M	Normal	0	−	>20	+	TND	D3
P3	45	M	Abnormal	120	+	216,396	+	15,216	D3

Abbreviations: PID, participant identification; CSF, cerebrospinal fluid; WBC, white blood cells; HBsAg, hepatitis B surface antigen; HBV, hepatitis B virus; DNA, deoxyribonucleic acid; IU/mL, international units per milliliter; TND, target not detected; M, male; +, positive; −, negative. Note: HBV DNA was quantified using the COBAS TaqMan HBV Test, version 2.0, in an earlier study with a reported lower limit of detection of 20 IU/mL. Samples with no detectable HBV DNA signal were reported as target not detected (TND). To account for dilution, the measured HBV DNA concentrations were multiplied by 2 for 1:1 dilutions and by 3 for 1:2 dilutions as described in the previous study [14].

**Table 2:** Clinically relevant HBV mutations identified in plasma and CSF sequences.

Sample	Genotype	Drug Resistance	Vaccine Escape	HCC-Associated
P1_PL	A1	None	None	T1858C
P1_CSF	A1	None	None	None
P2	D3	None	None	BCP_double_mutation (A1762T/G1764A), G1896A, G1899A
P3	D3	rtS184L, rtF194L, rtP236T	sP127L, sQ129L, sM133L sS136P	None

Abbreviations: CSF, cerebrospinal fluid; PL, plasma; HCC, hepatocellular carcinoma; BCP, basal core promoter; rt, reverse transcriptase; s, surface protein; P, participant; A1, HBV genotype A1; D3, HBV genotype D3.

## Data Availability

The raw data supporting the conclusions of this article will be made available by the authors on request.

## Abbreviations

The following abbreviations are used in this manuscript:

ADV	Adefovir
AHD	Advanced HIV Disease
BBB	Blood–Brain Barrier
BCP	Basal Core Promoter
CM	Cryptococcal Meningitis
CNS	Central Nervous System
CSF	Cerebrospinal Fluid
DNA	Deoxyribonucleic Acid
ETV	Entecavir
HBeAg	Hepatitis B e Antigen
HBsAg	Hepatitis B Surface Antigen
HBV	Hepatitis B Virus
HCC	Hepatocellular Carcinoma
HIV	Human Immunodeficiency Virus
LAM	Lamivudine
LdT	Telbivudine
MHR	Major Hydrophilic Region
NTCP	Sodium Taurocholate Co-Transporting Polypeptide
pgRNA	Pregenomic RNA

## Appendix A

**Table A1:** Baseline HBV markers and amplification outcome.

Sample No.	Subject_Identifier	HBsAg	HBV_VL	Sample_Type	Amplification Success
1	P1_CSF	pos	142,192	csf	Yes
2	P1_PL	pos	170,000,000	pl	Yes
3	P2_CSF	pos	TND	csf	Yes
4	P2_PL	neg	20	pl	No
5	P3_CSF	pos	15,216	csf	Yes
6	P4_CSF	pos	TND	csf	No
7	P5_CSF	pos	3060	csf	No
8	P5_PL	-	170,000,000	pl	No
9	P6_PL	pos	20	pl	No
10	P6_CSF	pos	TND	csf	No
11	P7_CSF	pos	942,153	csf	No
12	P7_PL	pos	-	pl	No
13	P8_CSF	neg		csf	No
14	P9_CSF	pos	20	csf	No
15	P9_PL	pos	20	pl	No
16	P10_CSF	neg	20	csf	No
17	P10_PL	pos	1512	pl	No

Abbreviations: CSF, cerebrospinal fluid; PL, plasma; HBsAg, hepatitis B surface antigen; HBV, hepatitis B virus; VL, viral load; TND, target not detected; P, participant; pos, positive; neg, negative.
